# Pituitary Society Delphi consensus on the role of radiotherapy for pituitary adenomas

**DOI:** 10.1007/s11102-026-01759-6

**Published:** 2026-09-23

**Authors:** Frederic Castinetti, Cecilia Piazzola, John Ayuk, Michael Kosmin, Marco Losa, Hani J. Marcus, Alberto M. Pereira, Shlomo Melmed, Maria Fleseriu, Pituitary Society Radiation Therapy for Pituitary Adenomas Delphi Consortium, Tushar Bandgar, Bettina Biagetti, Nienke Biermasz, Philippe Chanson, Daniela Esposito, Hidenori Fukuoka, Monica Gadelha, Monica Livia Gheorghiu, Sheryl Green, Mark Gurnell, Jose Miguel Hinojosa-Amaya, John A. Jr. Jane, Niki Karavitaki, Ann McCormack, Pietro Mortini, Lisa Nachtigall, Maria M. Pineyro, Jean Regis, Olabisi Sanusi, Jason Sheehan

**Affiliations:** 1 UMR 1251, Department of Endocrinology, Aix Marseille Université, INSERMMarMaRa InstituteAPHM, La Conception University Hospital, Marseille, MMG France; 2 Department of Endocrinology, University Hospitals Birmingham NHS Foundation Trust, Birmingham, UK; 3 Department of Radiotherapy, University College London Hospitals NHS Foundation Trust, and NIHR University College London Hospitals Biomedical Research Centre, London, NW1 2BU UK; 4 Department of Neurosurgery, IRCCS San Raffaele Scientific Institute, Vita-Salute University, Milan, Italy; 5 Department of Neurosurgery, National Hospital for Neurology and Neurosurgery, Victor Horsley, UCL Queen Square Institute of Neurology, University College London, London, UK; 6 Department of Endocrinology and Metabolism, Amsterdam University Medical Center, Amsterdam, The Netherlands; 7 Pituitary Center, Dept of Medicine, Cedars-Sinai Health Sciences University, Los Angeles, CA USA; 8 Pituitary Center, Departments of Medicine and Neurological Surgery, Oregon Health & Science University, Portland, OR USA

**Keywords:** Radiotherapy, Acromegaly, Cushing's disease, Pituitary adenoma, Surgery

## Abstract

**Context:**

Radiotherapy is used as second- or third-line treatment after transsphenoidal surgery for both functioning and nonfunctioning pituitary adenomas. Improved precision delivery may reduce side effects, but use overall has declined, partly due to improved surgical technique and advances in medical therapy, partly reflecting a shift from fractionated radiotherapy to stereotactic radiosurgery in appropriately selected patients, and also due to lack of clear, evidence-based guidelines. Questions remain regarding radiotherapy indications, optimal timing, choice of technique, and follow-up for efficacy and side effects. The aim of this Delphi panel was to establish expert consensus in these areas.

**Design:**

The Pituitary Society Steering Committee conducted a modified two-round Delphi panel with 29 international experts from five continents, including neuroendocrinologists, neurosurgeons and radiation oncologists.

**Results:**

Among the 26 initial statements, 22 (84.6%) reached agreement after Round 1. Four statements were reconsidered in Round 2, together with an additional question, after which 24 of 27 (88.9%) reached consensus. Panelists agreed that radiotherapy indication and timing should be assessed by expert multidisciplinary tumor boards, including a radiation oncologist, with the technique determined according to patient and adenoma characteristics. They emphasized controlling hormonal secretion while waiting for maximal radiotherapy efficacy in functioning adenomas. Long term follow-up > 10 years is required to assess for delayed efficacy, recurrence and side effects, although panelists agreed on the relatively low medium-term risk with modern techniques.

**Conclusion:**

Modified Delphi panel provides practical guidance regarding indications, timing, choice of technique and long-term follow-up of radiotherapy in pituitary adenomas. Modern techniques appear as effective and potentially safer vs. previous ones. Further long-term comparative data are needed.

## Introduction

Pituitary adenomas are benign tumors classified by size, transcription factors and hormonal secretion, either as functioning or non-functioning [1]. The first-line treatment is usually transsphenoidal surgery which induces remission in 40–95% of functioning pituitary adenomas [2–6] depending on adenoma’s subtype and characteristics [7–11]. In non-functioning pituitary adenomas, radiotherapy may be considered as a second line treatment after unsuccessful transsphenoidal surgery, particularly for large residual adenoma, remnant growth or recurrence. In functioning pituitary adenomas, radiotherapy can also be considered a second or a third-line treatment when medical treatment is poorly tolerated or ineffective [12]*.*

Radiotherapy modalities vary in how they deliver radiation to tissue, and in how precisely the radiation is limited to the target zone [13]. Despite technical improvements, radiotherapy use is declining in registries [14–18]. This is especially true for functioning pituitary adenomas, in which effective and well-tolerated medical treatments are accessible, and new delivery forms have improved patient satisfaction [8–10]. Interestingly, radiotherapy use has also decreased for non-functioning pituitary adenomas, despite repeat surgery, the only other treatment option, not always being feasible or effective, particularly following previous surgery in an expert center [19]. The decline has not been uniform across modalities, with stereotactic radiosurgery increasingly replacing fractionated radiotherapy when adenoma size and distance from the optic apparatus are favorable.

Although existing guidelines address radiotherapy in selected clinical settings, uncertainty remains regarding up-to-date indications, timing, technique selection and long-term follow-up. The Pituitary Society convened a Steering Committee which designed a modified Delphi panel to establish contemporary multidisciplinary expert consensus regarding the role of radiotherapy in pituitary adenomas. In this manuscript, the term “radiotherapy” is defined as a modality using radiation to treat a pituitary adenoma: this includes fractionated radiotherapy, stereotactic radiosurgery, and proton beam therapy.

## Methods

### Delphi panelists

The Pituitary Society nominated FC to chair the steering committee (SC), which was composed of 7 additional international experts (JA, MF, MK, HM, ML, SM, AMP). The steering committee identified eligible panelists and helped define the questions. Eligible panelists were endocrinologists, neurosurgeons or radiation oncologists, members of the Pituitary Society who were particularly involved in care and follow-up of patients with pituitary adenomas and who were recognized experts in this field based on PubMed publications over the last 10 years. Twenty-two were invited via email, of whom 21, from 15 countries accepted. Members of the SC also completed the surveys, resulting in a total panel of 29 experts. The SC and panelists were not compensated for their involvement.

### Study design

The principles of the modified Delphi panel are to propose several assertions (regarding use of radiotherapy in pituitary adenomas) to a group of experts from different specialties in an attempt to achieve consensus. This approach was chosen because several clinically important questions are supported mainly by retrospective evidence and expert opinion, with limited prospective comparative data.

The study comprised two successive rounds of anonymous online surveys with controlled feedback between rounds. The initial survey included 26 statements, for which panelists selected responses from predefined answer options or on a 5-point Likert scale (1 —Strongly Disagree, 2—Disagree, 3—Neutral, 4—Agree, and 5—Strongly Agree).

- **Round 1 statements development**

Questions were prepared by the SC, and the first round was open from February 16th to March 23rd, 2026. Statements were grouped into several overarching categories: general use of radiotherapy, indication of radiotherapy for functioning and non-functioning pituitary adenomas, long-term follow-up of radiotherapy for efficacy and side effects.

- **Round 2 statements development**

Round 2 was open from April 19th to June 7th, 2026. Statements reaching consensus in Round 1 were removed from Round 2. The SC evaluated Round 1 statements that had not reached consensus and, taking into account panel feedback, either rephrased or expanded before resubmission. An additional exploratory question was added following review of emerging literature on sex differences in the use of radiotherapy for pituitary adenomas.

### Statistical analyses

Consensus was defined as at least 70% of panelists choosing “strongly agree/agree” or “strongly disagree/disagree” for Likert scale statements. For questions with predefined categorical responses, consensus was defined as at least 70% of panelists choosing the same response [20–22]. Results were analyzed using the web-based platform EU Survey and Microsoft Excel.

## Results

Twenty-six statements were included in Round 1, of which 22 (84.6%) reached agreement. The remaining four statements were then proposed for a second round, and one additional exploratory question on sex differences in the use of radiotherapy for pituitary adenomas. After Round 2, 24 of the 27 statements considered across both rounds had reached consensus (Table 1). These statements are presented in 4 different areas: general use of radiotherapy, radiotherapy in non-functioning pituitary adenomas, radiotherapy in functioning pituitary adenomas, and long-term follow-up.

**Table 1:** List of statements proposed to the panelists and outcomes of the rounds

N	Statement	Consensus	Agreements (%)	Round of consensus
1	The decision to use radiotherapy should be made by a multidisciplinary tumor board that includes an expert radiation oncologist, a neurosurgeon, and an endocrinologist, preferably within a PTCOE or equivalent center	Yes	97%	Round 1
2	The choice of radiation modality should be determined by an expert radiation oncologist within a multidisciplinary team based on patient and tumor characteristics	Yes	81%	Round 1
3	Stereotactic radiosurgery should be preferred for patients with small-to moderate volume well-delineated lesions, taking into account the distance from the optic apparatus	Yes	87%	Round 1
4	Hypofractionated or conventional fractionated radiotherapy should be preferred for patients with large tumor remnants, optic pathway encasement, multifocal disease or when stereotactic radiosurgery dose constraints cannot be safely achieved	Yes	84%	Round 1
5	Proton beam therapy might be considered, where available, to reduce off-target irradiation and late effects, especially in children, teenagers and young adults	Yes	81%	Round 1
6	The decision to use radiotherapy should consider patient factors (age and preferences), tumor factors (type, size, location relative to critical structures, invasiveness and biological aggressiveness), prior and alternative treatments (surgery and medical therapy), and the long-term treatment consequences (particularly hypopituitarism)	Yes	90%	Round 1
7	Radiotherapy should not be used as primary treatment for nonfunctioning pituitary adenomas in patients who are suitable surgical candidates	Yes	87%	Round 1
8	Radiotherapy may may be considered as the primary treatment for patients with nonfunctioning pituitary adenomas who are not suitable surgical candidates or refuse surgery	Yes	84%	Round 1
9	Radiotherapy should be considered as second-line treatment for patients with residual or recurrent nonfunctioning pituitary adenomas following surgery	Yes	72%	Round 1
10	Radiotherapy should be considered for patients with functioning and nonfunctioning pituitary adenomas if residual tumor demonstrates progression after surgery	Yes	80%	Round 1
11	For patients with functioning adenomas, radiotherapy may be considered in patients who are intolerant of medical therapy after surgery	Yes	87,5%	Round 1
12	Patients with functioning adenomas should be prescribed medications proven to achieve biochemical remission before starting radiation treatment, due to the delayed maximal antisecretory effect of radiotherapy	Yes	72%	Round 1
13	For patients with functioning adenomas, radiotherapy may be considered in patients whose disease is medically controlled in order to avoid lifelong medical therapy	Yes	73.3%	Round 1
14	Somatostatin receptor ligands be stopped one-to-three month prior to stereotactic radiosurgery	Yes	74%	Round 2
15	*Growth hormone receptor antagonists (for acromegaly) and adrenal steroidogenesis inhibitors (eg osilodrostat in Cushing’s disease) may enhance radiotherapy efficacy*	*No*		
16	For patients with non-aggressive adenomas on pathology, radiotherapy should be considered at the time of tumor progression	Yes	75.9%	Round 1
17	For patients with aggressive adenomas on pathology, radiotherapy may be considered immediately after surgery	Yes	78%	Round 1
18	The expected long-term tumor volume control rate following radiotherapy for pituitary adenomas is more than 75% at 10 years	Yes	72%	Round 1
19	An increase in tumor volume after radiotherapy should raise concern for transformation to an aggressive pituitary adenoma	Yes	71,5%	Round 1
20	Remission of hormone hypersecretion should be monitored after radiotherapy in patients with functioning adenomas for more than 10 years	Yes	75%	Round 1
21	After radiotherapy for patients with nonfunctioning pituitary adenomas, a pituitary MRI should be performed regularly for more than 10 years	Yes	81%	Round 1
22	Biochemical screening for hypopituitarism should be performed for more than 10 years after radiation therapy	Yes	78%	Round 1
23	Risk of radiation-induced optic neuropathy is estimated to be less than 2%	Yes	72%	Round 1
24	Risk of radiation-induced secondary brain tumors is estimated to be less than 5%	Yes	100%	Round 2
25	*In patients who have received pituitary radiotherapy, brain imaging should only be performed if new clinical symptoms or signs arise*	*No*	*No consensus*	
26	There is currently insufficient evidence on the long-term risk of neurocognitive impairment and stroke with modern radiotherapy techniques	Yes	78.5%	Round 1
27	*In your clinical practice patient sex affects your decision when considering treatment with radiotherapy?*	*No*	*No consensus*	

### General use of radiotherapy

**Box 1: General use of radiotherapy (Statements 1, 2, 3, 4, 5, 6, 10, 16 and 17)**

**Table:** 

• The decision to use radiotherapy should:
○ Be made by a multidisciplinary tumor board including an expert radiation oncologist, neurosurgeon, and an endocrinologist, preferably within a Pituitary Tumor Center of Excellence (PTCOE), or equivalent
○ Consider patient factors (age and preferences), adenoma type, size, location and relation to critical structures, invasiveness and biological aggressiveness; prior and alternative treatments (surgery and medical therapy), and long-term treatment consequences (particularly hypopituitarism)
• Radiotherapy should be considered
○ At the time of adenoma progression for patients with non-aggressive adenomas on pathology
○ Immediately after surgery for patients with aggressive adenoma pathology
○ If residual adenoma tissue demonstrates progression after surgery for functioning and nonfunctioning pituitary adenomas
• The choice of radiation modality should be determined by an expert radiation oncologist within a multidisciplinary team based on patient and adenoma characteristics
○ Stereotactic radiosurgery is favored for patients with small to moderate volume well-delineated lesions, taking into consideration the distance from the optic apparatus
○ Hypofractionated or fractionated conformal radiotherapy should be favored for patients with large adenoma remnants, optic pathway encasement, multifocal disease or when stereotactic radiosurgery dose constraints cannot be safely achieved
○ Proton beam therapy might be considered, where available, to reduce off-target irradiation and late effects, especially in children, teenagers and young adults

When considering the use of radiotherapy in patients with pituitary adenomas, both the need for radiotherapy and its optimal timing should be decided by a multidisciplinary tumor board. Several factors to be considered include patient’s age and preferences, adenoma size and behavior, proximity to critical structures, and the availability, efficacy and tolerability of alternative medical treatments. The optimal timing of radiation for pituitary adenomas considered pathologically aggressive remains unclear as recently described in the revised ESE guidelines on aggressive pituitary tumors and carcinomas [23]. However, the optimal interval was not explored further in this Delphi either.

After radiotherapy has been agreed, an expert radiation oncologist should decide the most appropriate technique based on adenoma size and definition, prior invasion on imaging, proximity to the optic chiasm, and side effect risk.

Table 2 summarizes the different modalities of pituitary radiotherapy [13, 24–43].

**Table 2:** Different modalities of radiation techniques for pituitary adenomas

Name of the technique	Fractionated or single dose	Specific aspects	Mechanism of efficacy	Efficacy
Fractionated external beam radiotherapy	Fractionated	Best known fractionated technique	The target zone is destroyed due to the phenomenon of cellular DNA damage and repair with DNA damage induced by the procedure repaired more rapidly in healthy cells than in tumor cells, leading to adenoma destruction due to repeated radiotherapy session	Anti-tumor efficacy: superior to 90% Antisecretory efficacy: up to 70%
3D-conformal radiotherapy	Fractionated	Improvements in 3D planning vs Fractionated external beam radiotherapy		
Hypofractionated stereotactic radiotherapy	Fractionated	Non-invasive, repositionable stereotactic frame		
Gamma Knife radiosurgery	Single session	Stereotactic headframe	The target zone is destroyed due to the high dose, which is delivered very precisely	Anti-tumor efficacy: superior to 90% Antisecretory efficacy: 50–60%
CyberKnife	Single or low number of sessions	Robotic arm, patient immobilization with a thermoplastic mask, and real-time analysis of information	The target zone is destroyed due to the high dose, which is delivered very precisely	Anti-tumor efficacy: superior to 90% Antisecretory efficacy: 50–60%
Proton beam	Fractionated	Beam of high energy protons	Dose of high energy protons precisely targeted at a tumor, reducing the damage to surrounding healthy tissues and vital organs	Limited literature data in pituitary adenomas

#### Non-functioning pituitary adenomas

**Box 2: Radiotherapy in non-functioning pituitary adenomas (Statements 7, 8 and 9)**

**Table:** 

• Radiotherapy should not be used as primary treatment for nonfunctioning pituitary adenomas in patients who are suitable surgical candidates
• Radiotherapy should be considered:
○ as primary treatment for patients with nonfunctioning pituitary adenomas who are not suitable surgical candidates or decline surgery
○ as second-line treatment for patients with residual or recurrent nonfunctioning pituitary adenomas following surgery

Transsphenoidal surgery is the first line treatment of non-functioning pituitary adenomas. Medical treatment is of limited efficacy in controlling adenoma volume [44]. When patients requiring intervention cannot be operated or decline surgery, radiotherapy is the sole alternative option. Several studies and systematic reviews have demonstrated high rates of adenoma growth control following radiotherapy [24, 27–29, 45–48]. As the decision for surgery usually implies a macroadenoma threatening the optic apparatus, non-fractionated radiation techniques should not be used due to the risk of radiation-induced optic neuropathy [30, 49]. Moreover, in extensive adenomas or those invading the cavernous sinuses, fractionated conformal radiotherapy is preferred [29]. After incomplete surgery, radiation treatment is considered for growing adenoma remnants. The expert radiation oncologist selects the most appropriate technique according to adenoma size, definition and relationship to critical structures.

#### Functioning pituitary adenomas

**Box 3: Radiotherapy in functioning pituitary adenomas (Statements 11, 12, 13 and 14)**

**Table:** 

• For patients with functioning adenomas, radiotherapy may be considered:
○ in patients whose disease is medically controlled in order to avoid lifelong medical therapy
○ in patients who are intolerant of medical therapy after surgery
○ in patients uncontrolled on medical therapy despite maximal tolerated doses
• Patients with functioning adenomas should be prescribed medications to achieve biochemical remission before starting radiation, due to the delayed maximal antisecretory effect of radiotherapy
• Pituitary directed drugs (somatostatin receptor ligands and/or dopamine agonists) should be discontinued one-to-three-month prior to stereotactic radiosurgery when feasible

Transsphenoidal surgery is the first-line treatment for functioning pituitary adenomas (except for prolactinomas where medical treatment is first line for most patients) [50]. Recent guidelines for management of acromegaly, Cushing’s disease and prolactinoma suggested that radiotherapy should be considered a second or third-line treatment after transsphenoidal surgery and medical treatments [50–52]. Several studies and systematic reviews reported varying radiotherapy efficacy for functioning pituitary adenomas [25, 31, 33, 53–61]. Radiotherapy has also been considered to possibly avoid long-term use of medical treatments in selected patients whose disease is otherwise well controlled, or in patients where medical therapy is poorly tolerated. Given the delay in achieving maximal anti-secretory efficacy (up to 10 years), panelists agreed on the need for an effective antisecretory treatment before and after radiotherapy [24, 27–29, 45–48]: enabling secretion control while awaiting radiotherapy efficacy.

The impact of antisecretory treatments on radiation efficacy has long been debated for Gamma Knife radiosurgery; the hypothesis being that decreased adenoma cell proliferation while on medical treatment would decrease radiation efficacy. Since the initial study published by Landolt et al., several retrospective series have been published with contradictory results for acromegaly (somatostatin receptor ligands) and prolactinoma (dopamine receptor agonists) [62–64]. In view of this uncertainty and the underlying biological rationale, the panel supported temporary withdrawal of somatostatin receptor ligands and dopamine agonists before stereotactic radiosurgery, where this can be done safely and with timing guided by the pharmacokinetic properties of the agent.

### Long-term follow-up

**Box 4: Long-term follow-up of radiotherapy (Statements 18, 19, 20, 21, 22, 23, 24 and 26)**

**Table:** 

• Expected long-term volume control rate following radiotherapy for pituitary adenomas is > 75% at 10 years
• After radiotherapy for patients with nonfunctioning pituitary adenomas, pituitary MRI should be performed regularly for > 10 years
• Increased adenoma volume after radiotherapy should raise concern for transformation to an aggressive pituitary adenoma
• In patients with functioning adenomas remission of hormone hypersecretion should be monitored after radiotherapy for > 10 years
• Biochemical screening for hypopituitarism should be performed for > 10 years after radiation therapy
• Risk of radiation-induced optic neuropathy is < 2%
• Risk of radiation-induced secondary brain tumors is < 5%
• There is insufficient evidence on the long-term risk for neurocognitive impairment and stroke with modern radiotherapy techniques

Adenoma growth control is high with all contemporary radiotherapy techniques [24, 27–29, 45–48]. More than 75% of adenomas remain stable or decrease in size, however a small proportion progress [65]. Where growth occurs, it is important to consider whether the treated mass was adequately covered or whether growth represents clinically aggressive behavior [66]. Anti-tumor efficacy usually optimizes within 3 years [24, 27–29, 45–48], and the panel agreed on performing MRI for at least 10 years to ensure remnant stability.

Anti-secretory efficacy varies widely and depends on the initial hormonal level and radiation technique. The timeline usually depends on the technique, with a mean of 2–5 years with focused radiation techniques, and slightly more for fractionated techniques. Hormone levels decline progressively requiring regular drug withdrawal during follow-up to determine whether radiotherapy was effective in suppressing excess hormone secretion [25, 31, 33, 53–61]. Moreover, up to 20% recur with focused radiation techniques during long-term follow-up [53]. Experts agreed to monitor hormonal hypersecretion for at least 10 years, even in patients presumed to be in remission post radiotherapy, to assess for recurrence.

The risk of side effects has been well studied for older techniques and pituitary radiotherapy has been associated with increased mortality (particularly cerebrovascular disease) [67]. However, long-term risk data are lacking for the more modern radiation techniques. The risk of hypopituitarism increases with time and is maximal within 10 years after radiation [24, 34, 48, 55, 68]. In a retrospective UK study there was a modest (< 5%) increased risk for brain tumors in patients undergoing radiotherapy [69]. Contradictory results had been shown by others [70–74]. In particular, case series’ of single-fraction stereotactic radiosurgery with long-term imaging follow-up have not reported increased rates of radiation-associated intracranial tumors as compared to expected population rates. The risk of radiation-induced optic neuropathy mainly concerns focused radiation techniques: a low dose to the optic chiasm should decrease this risk, now estimated to be < 2% [49]. There has been no very-long term study characterizing risks of cognitive side effects with modern radiation techniques, clearly described with older ones [75, 76].

### Statements without agreement (Statements 15, 25, 27)

**Table:** 

• “There is no clear evidence that growth hormone receptor antagonists and adrenal steroidogenesis inhibitors enhance radiotherapy efficacy.”
• “In patients who have received pituitary radiotherapy, brain imaging should be performed if new clinical symptoms or signs arise”
• “In your clinical practice patient sex affects your decision when considering radiotherapy.”

As pituitary-targeting drugs have been proposed to impair radiation efficacy, some have suggested that peripheral-targeted treatments as growth hormone receptor antagonists and steroidogenesis inhibitors, might potentiate efficacy by increasing pituitary proliferation. These may lead to moderate adenoma growth in ~ 10% of patients during follow-up [77, 78]. It remains uncertain whether growth is due to the natural adenoma history, or to indirect medication effect with decreased feedback suppression. No agreement was reached on potential benefits of pre-treating with peripherally acting drugs to improve radiation efficacy.

While radiation treatment increases the risk of stroke and brain tumors (in < 5% [79, 80]), there was no agreement on the need to systematically perform a brain MRI during follow-up. Some considered that regular imaging follow-up should be performed, irrespective of suggestive clinical signs. However, no time interval between each imaging was agreed upon.

A recent review on the sex difference in radiotherapy efficacy and toxicity showed that sex is not a predictive factor for either of them [81]. However, some experts considered that the risk of pituitary deficiency might be more impactful for women of childbearing age than for men.

## Discussion

While radiotherapy exhibits anti-secretory and anti-tumor efficacy, its use has declined over 20 years [14], likely due to the availability of highly effective surgical and medical treatments, as well as continuing concerns regarding long-term safety. The overall decline also reflects a change in the modality used, with stereotactic radiosurgery increasingly replacing fractionated radiotherapy when adenoma size and distance from the optic apparatus are favorable. These improved stereotactic approaches may lead to a reevaluation of the role of modern radiation techniques in the management of pituitary adenomas. Recent guidelines on acromegaly, Cushing’s disease, prolactinoma, pituitary incidentaloma, and aggressive pituitary adenomas [23, 50–52, 82], all briefly define these roles, after unsuccessful surgery or medical treatment, or when a post-surgical tissue remnant grows, especially with aggressive adenomas (Table 3). The aim of this modified Delphi panel was to further clarify further and elucidate the role of radiotherapy, and its different modalities in the management of pituitary adenomas.

**Table 3:** Brief discussion of radiotherapy role reviewed in the most recent guidelines on functioning and non-functioning pituitary adenomas

Guidelines	1 st author, Year	Discussion of radiotherapy role in each recent pituitary adenomas guidelinesand consensus statements
ESE revised guidelines on aggressive pituitary tumours and carcinoma (European Society of Endocrinology)	Raverot, 2025 (Ref#23)	We recommend radiotherapy (RT) to improve tumour control in patients with clinically relevant tumour progression despite surgery and standard medical treatment We suggest adjuvant radiotherapy, typically 3–6 months following surgery, be considered in the setting of a clinically relevant invasive tumour remnant with proliferation markers and/or genetic alterations, strongly indicating aggressive behaviour
Pituitary incidentaloma (Pituitary Society)	Fleseriu, 2025 (Ref#82)	No discussion of radiotherapy for pituitary adenomas
Consensus on acromegaly therapeutic outcomes (Acromegaly Consensus Group)	Melmed, 2025 (Ref#51)	Stereotactic radiosurgery or surgical intervention or reintervention should be considered, if control is not achieved with medical therapies
Diagnosis and management of prolactinoma (Pituitary Society)	Petersenn, 2023 (Ref#50)	Radiation therapy should be reserved for patients who show poor mass shrinkage in response to dopamine agonists and have either non-resectable residual adenoma tissue after surgery or contraindications for surgery (strong). Stereotactic radiotherapy techniques yield improved outcomes and have now become standard of care where available (strong). Patients should be advised that response to radiotherapy can take several years (strong). Patients should be informed about potential adverse effects occurring even many years after treatment and should be followed lifelong to detect hypopituitarism, optic neuropathy, cranial nerve palsy or second brain tumours (strong). Radiation therapy is the least used management approach for prolactinoma and is mainly offered when medical and surgical treatments have not been successful, usually in patients with size-progressing, aggressive prolactinoma or prolactin-secreting malignancies
Consensus on diagnosis and management of Cushing’s disease: a guideline update (Pituitary Society)	Fleseriu, 2021 (Ref#52)	Radiotherapy is most commonly used in cases of persistent hypercortisolism after incomplete corticotroph tumour resection, particularly if the tumour is aggressive or invasive or is considered unresectable (high quality, strong recommendation). Stereotactic radiosurgery is probably more convenient as few treatment sessions are required, but avoiding optic chiasm exposure is crucial (high quality, strong recommendation). Lifelong monitoring for pituitary hormone deficiencies and recurrence is required in all patients undergoing radiotherapy (high quality, strong recommendation). Imaging for secondary neoplasia in the radiation field should also be considered (high quality, strong recommendation)

Formal graded specific guidelines on radiotherapy use in pituitary adenomas would be challenging to produce given the low level of reported evidence. Prospective studies are challenging, as they require a large number of patients and a very prolonged follow-up to ascertain both long-term efficacy and side-effects, but plans for clinical trials are incipient. Thus, this modified Delphi panel has the advantage of combining international pituitary experts’ opinions to help define the contemporary use of radiotherapy.

The panel assertions were initially aimed at defining the role of radiotherapy as a whole, i.e. grouping together the treatment modalities using radiation. Subsequent questions asked more detail regarding the different approaches, specifying their roles, merits and pitfalls (Table 2).

First, the expert panel emphasized the need to at least consider radiotherapy for treating a pituitary adenoma after unsuccessful transsphenoidal surgery, either as a second or a third-line treatment. Adjuvant antisecretory efficacy is reported in 30–80% of patients [25, 31, 33, 53–61], and adenoma growth control is achieved in > 90% in reported series, with the panel agreeing on an expected control rate of > 75% over 10 years [24, 27–29, 45–48].

The decision to perform radiotherapy should be made by an expert multidisciplinary tumor board. Most non-aggressive pituitary adenoma remnants grow slowly. However, an expert neuroradiologist should compare subsequent pituitary MRIs with the initial post-operative MRI, to determine growth trajectories. The multidisciplinary tumor board will consider the need for radiotherapy after accounting for the efficacy of alternative treatments, such as medications, or a repeat surgery, as soon as the remnant is noted to grow.

The presence of an aggressive pituitary adenoma raises the question of optimal timing for radiotherapy. As emphasized in recent European guidelines [23], radiotherapy should be performed promptly after surgery, once approved by the multidisciplinary tumor board.

As detailed in Table 2, indications for more frequently employed stereotactic radiosurgery, and fractionated radiotherapy differ. The main factors to consider are the size and the location of the pituitary adenoma remnant. Although stereotactic radiosurgery is more convenient for the patient, it requires a very well-defined target visible on a pituitary MRI to minimize the risk of recurrence (reported in up to 20% of patients), and a predefined low dose to the chiasm (or a minimal distance to the chiasm) to avoid the risk of radiation-induced optic neuropathy (reported in less than 2% of patients) [83]. Both approaches have similar efficacy but the risk of subsequent hypopituitarism appears to be lower with stereotactic radiosurgery [32]. Importantly, stereotactic radiosurgery is not usually the first option for poorly defined large tissue remnants, or those located in both cavernous sinuses. The expert radiation oncologist plays a key role in determining the most suitable radiotherapy approach for each patient.

The Delphi panel emphasized the need for controlling hormonal excess while awaiting maximal radiotherapy efficacy in functioning adenomas. Prolonged follow-up (> 10 years) is mandatory to determine delayed efficacy, possible recurrence, and side effects. While the modified Delphi panel did not necessarily consider detailed side effect prevalence rates, the panel agreed that the medium-term risk of serious adverse effects with modern techniques appears relatively low, while acknowledging that long-term results are limited. The risk of secondary tumors may only manifest after decades and could at least partly explain the declined radiotherapy use.

Compared with the most recent guidelines (Table 3), [23, 50–52, 82], this consensus adds important details in several areas of radiation therapy use for pituitary adenomas. The recent Pituitary Society statement on incidentalomas does not discuss radiotherapy, and the panel includes explicit guidance for non-functioning adenomas. The panel here defines the choice of stereotactic radiosurgery or fractionated radiotherapy anatomically, according to the adenoma characteristics set out above. Neither the management of pituitary-directed medical therapy around stereotactic radiosurgery nor the role of proton beam therapy is addressed in current guidelines for hypersecreting pituitary adenomas, and both were considered here. Where guidelines recommend lifelong follow-up in general terms, here we specify which parameters should be monitored and for how long, and the panel attaches quantitative estimates to expected tumor control and to the risks of optic neuropathy and secondary brain tumors.

Unanswered questions include the need to evaluate the benefits and side effects of proton beam therapy for pituitary adenomas. The impact of adjuvant antisecretory treatment remains uncertain and warrants prospective study. There is also a need for multicenter long-term registries for evaluating side effects, and patients treated by modern modalities should ideally be offered inclusion in anonymized registries. Health-economic evaluation may more clearly define the role of radiotherapy for functioning adenomas who would otherwise require lifelong medical therapy [84].

Strengths of this Delphi survey include: the panel comprised of pituitary experts representing a wide geographical diversity across five continents, and its multidisciplinary nature including neurosurgeons, neuroendocrinologists, and radiation oncologists; the high response rate with limited panelist attrition occurring across rounds; and use of a web-based survey. Limitations include variation in practice between centers and countries, and possible selection bias arising from inclusion of Pituitary Society experts, whose clinical practice may include a greater proportion of recurrent or clinically aggressive adenomas. As for all Delphi studies, consensus reflects expert opinion rather than comparative evidence and should not replace individualized multidisciplinary decision-making. Comparisons between stereotactic radiosurgery and fractionated radiotherapy are also confounded by indication, since smaller, well-delineated adenomas at an adequate distance from the optic apparatus are preferentially selected for radiosurgery, whereas larger adenomas adjacent to the optic apparatus are treated with fractionated radiotherapy. Reported differences in rates of subsequent hypopituitarism and secondary tumor formation may therefore reflect case-mix as well as modality. Consistent with this, the panel defined modality selection by adenoma size and anatomy rather than ranking one technique above another.

This modified Delphi panel provides practical multidisciplinary guidance on the use of radiotherapy for treating pituitary adenomas. Radiotherapy is an important option for selected patients, with decisions regarding its use and delivery made within an expert multidisciplinary team and informed by the clinical context, adenoma characteristics and available alternatives. Modern radiotherapy techniques, and especially stereotactic radiosurgery, appear at least as effective as, and potentially safer than prior techniques; further long-term observation is however needed.

## Data Availability

No datasets were generated or analysed during the current study.
